# PIPE: a protein–protein interaction passage extraction module for BioCreative challenge

**DOI:** 10.1093/database/baw101

**Published:** 2016-08-14

**Authors:** Yung-Chun Chang, Chun-Han Chu, Yu-Chen Su, Chien Chin Chen, Wen-Lian Hsu

**Affiliations:** 1 Institute of Information Science, Academia Sinica, Taipei, Taiwan; 2 Department of Information Management, National Taiwan University, Taipei, Taiwan; 3 Department of Computer Science, National Tsing Hua University, Hsinchu, Taiwan

## Abstract

Identifying the interactions between proteins mentioned in biomedical literatures is one of the frequently discussed topics of text mining in the life science field. In this article, we propose PIPE, an interaction pattern generation module used in the Collaborative Biocurator Assistant Task at BioCreative V (http://www.biocreative.org/) to capture frequent protein-protein interaction (PPI) patterns within text. We also present an interaction pattern tree (IPT) kernel method that integrates the PPI patterns with convolution tree kernel (CTK) to extract PPIs. Methods were evaluated on LLL, IEPA, HPRD50, AIMed and BioInfer corpora using cross-validation, cross-learning and cross-corpus evaluation. Empirical evaluations demonstrate that our method is effective and outperforms several well-known PPI extraction methods.

**Database URL:**

## Introduction

Due to the rapidly growing number of research articles, researchers have found it difficult to retrieve the articles of their interest. To identify the specific ones that meet their requirements, biomedical researchers tend to leverage the relationship between entities mentioned in these publications. Among all types of biomedical relations, protein–protein interaction (PPI) has played a critical role in the field of molecular biology due to the increasing demands for the automatic discovery of molecular pathways and interactions from literature (1, 2). Understanding PPIs can help in predicting the function of uncharacterized proteins by distinguishing their role in the PPI network or comparing them to proteins with similar functionality (3). Additionally, composing networks of molecular interactions are useful in identifying functional modules or uncovering novel associations between genes and diseases. In essence, the ultimate goal of PPI extraction is to recognize various interactions including transcriptional and translational regulations, post translational modifications and dissociation between proteins within biomedical literature (4), so to find the criteria to judge whether a pair of proteins in the same sentence contains any interaction between them.

To extract PPIs from biomedical literatures in an effective manner, we present PIPE, a module named for PPI Pattern Extraction used in the Collaborative Biocurator Assistant Task (BioC) (5) at BioCreative V. The purpose of BioC is to create BioC (6)-compatible modules which complement one another and integrate them into a system that assists BioGRID curators. The track is divided into eight different subtopics and each subtask was addressed independently, including gene/protein/organism named entity recognition and protein-protein/genetic interaction passage identification. The mission of our team is to develop a module that can identify passages that convey interactions between protein mentions. To develop a PPI passage extraction module, we model PPI extraction as a classification problem and propose an IPT structure to represent syntactic, content and semantic information in text. The CTK is then adopted to integrate IPT with support vector machines (SVMs) to identify sentences referring to PPIs within biomedical literatures. Results of experiments demonstrate that the proposed CK method is effective in extracting PPI. In addition, the proposed interaction pattern generation approach successfully exploits the interaction semantics of text by capturing frequent PPI patterns. The method consequently outperforms the feature-based PPI method (7–10), the kernel-based PPI method (4, 11–13) and the shortest path-enclosed tree (SPET) (14) detection method, which are all widely used to identify relations between named entities. Our method also achieves comparable performances to those of multi-kernel-based methods (7, 15, 16).

The rest of the article is organized as follows. In ‘Related Work’ section, we review previous work, and briefly introduce kernel-based PPI methods. We describe the proposed PIPE system in ‘Methodology’ section. ‘Experiments’ section shows the experimental results and presents further comparison of our work with related work. Finally, we conclude our work in ‘Concluding Remarks’ section.

## Related work

Most PPI extraction methods can be regarded as supervised learning approaches. Given a training corpus containing a set of manually-tagged examples, a supervised classification algorithm is employed to train a PPI classifier to recognize whether an interaction exists in a sentence. Feature-based approaches and kernel-based approaches are frequently used for PPI extraction, where the former exploit instances of both positive and negative relations in a training corpus to identify effective text features. For instance, Landeghem *et al.* (8) proposed a rich-feature-based (RFB) method that applied feature vectors in combination with automated feature selection for PPI extraction. In addition, a co-occurrence-based method was introduced by Airola *et al.* (7), which explored co-occurrence features of dependency graphs for representing the sentence structure. However, feature-based methods often have difficulty in finding the effective features to extract entity relations (14). In order to address this problem, the kernel-based methods have been proposed to implicitly explore various features in a high dimensional space by employing a kernel to directly calculate the similarity between two objects (17). Formally, a kernel function is a mapping K:X×X→[0.∞): from input space *X* to a similarity score,

K(x,y)=ϕ(x)⋅ϕ(y)=∑iϕi(x)ϕi(y),

where ϕ_*i*_ (*x*) is a function that maps *X* to a higher dimensional space without needing to know its explicit representation. Such a kernel function makes it possible for us to compute the similarity between objects without enumerating all features, therefore reducing the burden of feature engineering on structured objects in Natural Language Processing (NLP) research, such as the tree structure in PPI extraction (18, 19). Examples include Erkan *et al.* (20) defining two kernel functions based on the cosine similarity and the edit distance among the shortest paths between protein names in a dependency parse tree; Satre *et al.* (9) also developed a system named AkanePPI, which extracted features using the combination of a deep syntactic parser to capture the semantic meaning of the sentences with a shallow dependency parser for the tree kernels. The latter enabled automatic generation of rules to identify pairs of interacting proteins from a training corpus.

The tree kernel-based method is widely used in PPI extraction due to its capability to utilize the structured information derived from sentences, especially for the constituent dependencies knowledge. Vishwanathan and Smola (12) proposed a subtree (ST) kernel which considered all mutual subtrees in the tree representation of two compared sentences. Here a ST comprised a node with all its descendants in the tree; two STs were considered identical if nodes in both STs had identical labels and order of their children. Likewise, Collins *et al.* (21) introduced a subset tree (SST) kernel that relaxed the constraint that requires all leaves to be included in the substructures at all times while preserving the grammatical rules; for any given tree node, either none or all of its children were included in the resulting subset tree. In addition, Moschitti (11) adopted a partial tree (PT) kernel which was more flexible by virtually allowing any tree sub-structures; the only constraint was that the order of child nodes must be identical. Both SST and PT kernels are CTKs. Kuboyama *et al.* (13) proposed a spectrum tree (SpT) kernel which emphasized the simplest syntax-tree substructures among these four tree kernels; it compared all directed vertex-walks, each of which represented by a sequence of edges connecting syntax tree nodes of length *q*. When comparing two protein pairs, the number of shared sub-patterns, or tree *q*-grams, were measured as the similarity score.

Current studies attempt to use multiple kernels to overcome the shortcoming of information loss of single kernel approaches. For instance, Miwaa *et al.* (16) proposed a composite kernel (CK) approach for PPI extraction that extracted and combined several different layers of information from a sentence with its syntactic structure by using several parsers. They outperformed other state-of-the-art PPI systems on four out of the five corpora because the combination of multiple kernels and parsers could gather more information and cover a certain fraction of the losses. In addition, Giuliano *et al.* (15) defined the Shallow Linguistic (SL) kernel as the sum of the global context and the local context kernel. For the global context kernel, the feature set was generated based on the position of words appearing in a sentence under three types of patterns (‘fore–between, between’ and ‘between–after’) relative to the pair of investigated proteins. Each pattern was represented using ‘bag of words’ as a term frequency vector; the global context kernel was in turn defined as the total count of mutual words in these three vectors. For the local context kernel, they utilized orthographic and SL features of sentences respect to the candidate proteins of the pair, of which the similarity was calculated using dot product. On the other hand, Airola *et al.* (7) integrated a parse structure sub-graph and a linear order sub-graph to develop the all-path graph kernel (GK). The former sub-graph represented the parse structure of a sentence and included words or link vertices; a word vertex contained its lemma and its parts-of-speech (POS), while a link vertex contained its link only. Both types of vertices possessed their positions relative to the shortest path. The linear order sub-graph represented the word sequence in the sentence. Thus, it accommodated word vertices, each of which contained its lemma, relative position to the target pair and POS. The experimental results demonstrated that their method is effective in retrieving PPIs from biomedical literatures.

The above discussion suggests that the hierarchical-structured features in a parse tree might not be fully utilized in previous work. On the other hand, we believe that the tree structure features could play a more important role than previously reported. Since convolution kernels (22) is capable of capturing structured information in terms of sub-structures (which provides a viable alternative to flat features), we therefore integrated the syntactic, content and semantic information of text into an interaction pattern tree structure to capture the sophisticated nature of PPIs. The concept is incorporated in PIPE to discriminate interactive text segments.

## 3. Methodology

**Figure 1.**
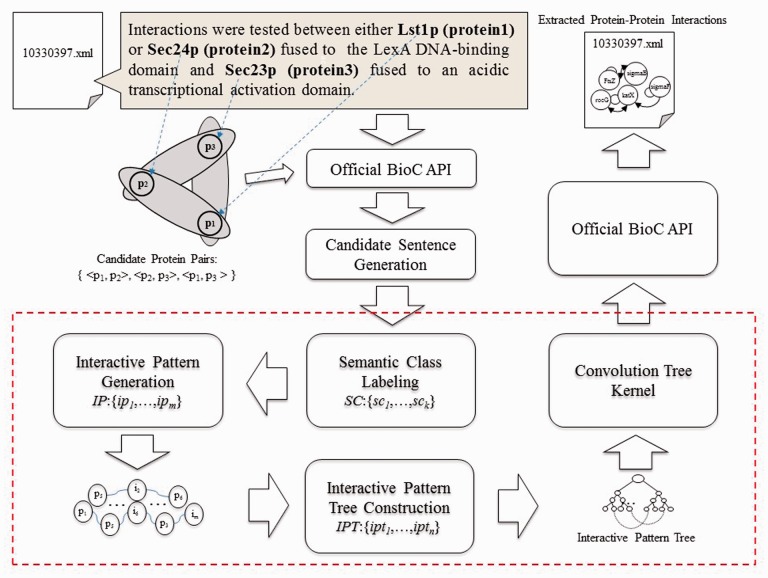
Overview of the PPI extraction method.

Figure 1 shows an overview of the proposed PPI extraction method, which is comprised of three key components: ‘interaction pattern generation, IPT construction’ and ‘CTK’. The paragraphs and related protein names in paragraphs are extracted from original XML files with the help of official BioC API, while candidate sentence generation produces a set of candidate sentences by capturing every sentence that contains at least two types of protein names. The candidate sentences then undergo the semantic class labeling (SCL) process, which help group together the synonyms. Since we treat PPI extraction as a classification problem, we use the interaction pattern generation component to automatically produce representative patterns for mentioned interactions between proteins. Subsequently, the IPT construction is used to integrate the syntactic and content information with generated interaction patterns for text representation. Finally, the CTK measures similarity between IPT structures via SVM to classify interactive expressions, followed by saving the results using official BioC API in XML format. Each component is elucidated in detail in the following sections.

### Candidate sentence generation

Our system is constructed with official BioC library. The API offers built-in functions for us to parse the documents as paragraphs and retrieve the annotations. It also provided function for separating sentences in a paragraph; nevertheless, to be able to use this function, the input XML files are required to place each sentence between specific tags. Since the example files for this sub-task do not possess such information, we have no other option but to come up with our own version of sentence splitter. To extract the sentences from the paragraphs that contain possible PPI, we retrieve each sentence that have at least two kinds of protein names. Since the names are specified with annotations and are already available with the metadata that comes with each paragraph, we can do a first-level filtering of each paragraph to see whether it contains over two different types of protein, with which the program proceeds if it does. We save the distinct protein names in a paragraph to a set PG.

**Figure 2.**
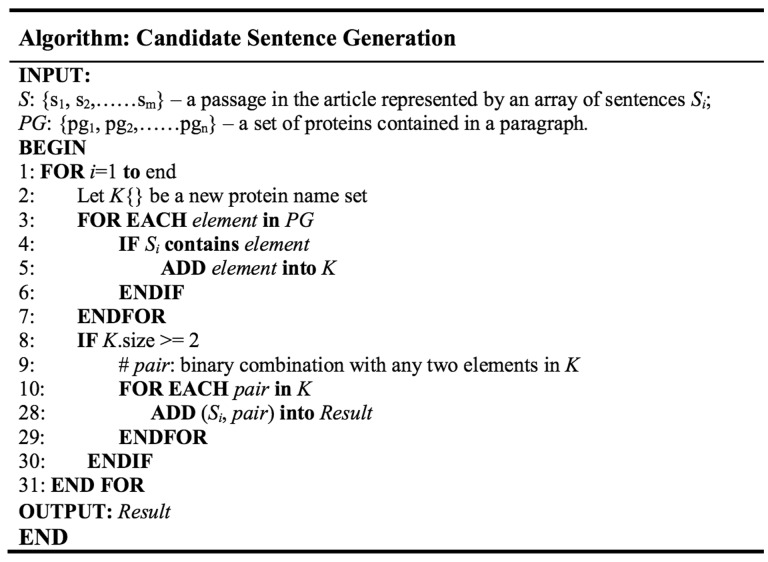
Candidate sentence generation algorithm.

**Figure 3.**
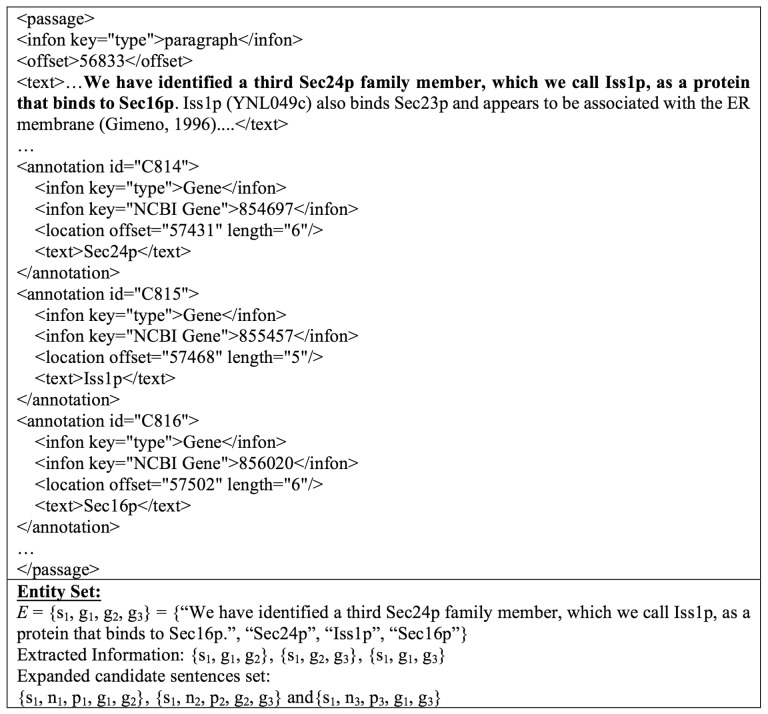
An example of expanded candidate sentence extraction.

Later on, we try to segment the sentences in a paragraph. Intuitively, using string splitting functions in programming language library with period as the separator seem like a good choice; unfortunately, bio-related documents tend to have periods used in purposes other than dividing sentences, such as in float number or abbreviation. As such, we took rule-based approach by inserting several conditions for the program to ignore the period under certain situations. Examples include neglecting period in Figure 3, ‘ph 6.5’, ‘Lin *et al.*’ etc. After each paragraph has been broken down, we save the sentences into a set *S *= {*s*_1_,…,*s_i_*}, where each sentence is denoted as *S_i_*. Each sentence is tokenized to find the protein names, since most protein names exist in the form of unigram. For protein names that contain spaces within, substring match of the whole sentence is used. In cases where there exist more than one type of protein in a single sentence, a function is used to list all protein names contained in a sentence and save them into a temporary set *K*. The program would then generate all possible pair-wise protein combinations, each of which will be attached at the end of the original sentence to produce a ‘candidate sentence’. The algorithm is illustrated in Figure 2. Output is then written to a text file, each line of which consist of the original sentence and two protein names.

**Figure 4.**
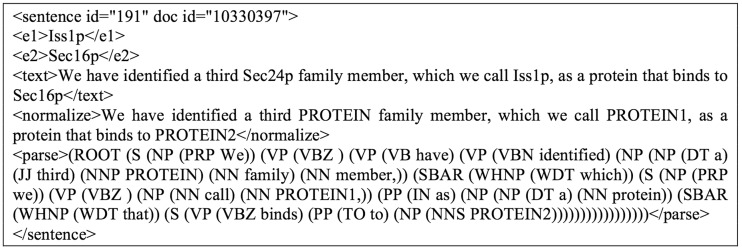
An example of the expanded candidate sentence {s1, n2, p2, g2, g3}.

Provided with the generated candidate sentences, we further process them with normalization and parsing before feeding them to the next step; normalization replaces all protein names found in a sentence with the label of “PROTEIN”, whereas parsing identifies the POS for each term. For instance, the sentence ‘We have identified a third Sec24p family member, which we call Iss1p, as a protein that binds to Sec16p’ contains recognized genes ‘Sec24p’, ‘Iss1p’ and ‘Sec16p’; thus we obtain a corresponding entity set *E* = {s_1_, p_1_, p_2_, p_3_} = {‘We have identified a third Sec24p family member, which we call Iss1p, as a protein that binds to Sec16p.’, ‘Sec24p’, ‘Iss1p’, ‘Sec16p’}, which could produce candidate sentences {s_1_, p_1_, p_2_}, {s_1_, p_2_, p_3_}, {s_1_, p_1_, p_3_}. The corresponding normalized sentences (*n_i_*) and parsed sentences (ϼ_*j*_) for an original sentence *s_i_* are added to its candidate sentence to form ‘expanded candidate sentences’; in this case, we get {s_1_, n_1_, ϼ_1_, p_1_, p_2_}, {s_1_, n_2_, ϼ_2_, p_2_, p_3_} and {s_1_, n_3_, ϼ_3_, g_1_, g_3_}, as illustrated in Figure 3. To explore in more detail, the content of the expanded candidate sentence set {s_1_, n_2_, ϼ_2_, p_2_, p_3_} is shown in Figure 4 as an example. The data are now ready for SCL.

### Learning interaction pattern from biomedical literature

The human perception of a PPI is obtained through the recognition of important events or semantic contents to rapidly narrow down the scope of possible candidates. For example, when an expression contains strongly correlated words such as ‘beta-catenin’, ‘alpha-catenin 57-264’ and ‘binding’ at the same time, it is natural to conclude that this is an expression of PPI, with a less likelihood of a non-interactive one. This phenomenon can be used to explain how humans can skim through an article to quickly capture the interactive expression. In light of this rationale, we propose an interaction pattern generation approach to automatically produce representative patterns from sequences of PPI expressions.

**Figure 5.**
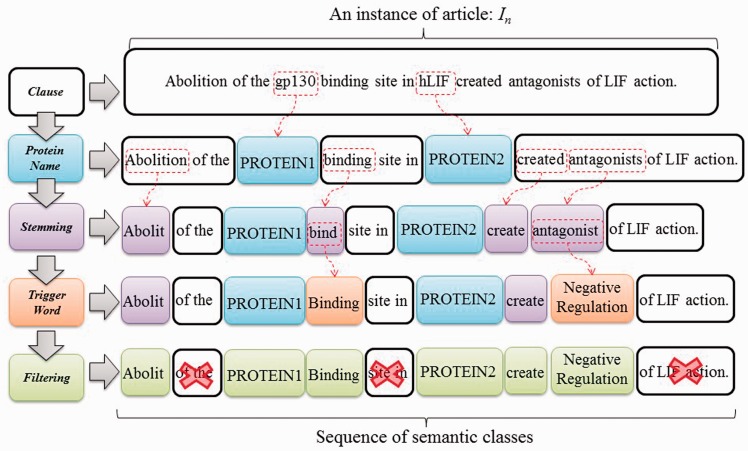
SCL process.

We formulate interaction pattern generation as a frequent pattern mining problem, starting by feeding the expanded candidate sentences sets obtained in the previous phase into SCL process. To illustrate the process of SCL, consider the instance *I_n _*= ‘Abolition of the gp130 binding site in hLIF created antagonists of LIF action’, as shown in Figure 5. ‘*gp130*’ and ‘*hLIF*’ are two given protein names first tagged as *PROTEIN1* and *PROTEIN2*, respectively. Remaining tokens are later stemmed using the porter stemming algorithm (23). Finally, trigger words ‘bind’ and ‘antagonist’ are labeled with their corresponding types by using our compiled trigger word list extracted from a BioNLP corpus (24). Evidently, the SCL can group the synonyms together through the same label. This enables us to find distinctive and prominent semantic classes for PPI expression in the following stage.

After SCs are introduced into the sequences, we construct a graph based on the co-occurrence of distinct SCs to describe the strength of relations between them. Since these sequences are of an ordered nature, the graph is directed and can be made with association rules. In order to avoid generating interaction patterns with insufficient length, we empirically set the minimum support of a SC to 20 and the minimum confidence to 0.5 in our association rules. [According to (25), rule support and confidence are two measures of rule value. Typically, association rules are considered valuable if they satisfy both a minimum support threshold and a minimum confidence threshold. Therefore, in our article, we set the minimum support of a SC to 20; i.e. we only consider SCs whose occurring frequency are more than 20.] This setting is derived from the observation that the rank-frequency distribution of SCs followed Zipf’s law (26), therefore does the normalized frequency of interaction patterns. SCs with lower frequencies are generally irrelevant to PPI. For that reason, we select the most frequent occurring SCs with accumulated frequencies exceeding 70% of the total SC frequency count in the positive PPI sentences. An association rule is represented as Equation (1).

**Figure 6.**
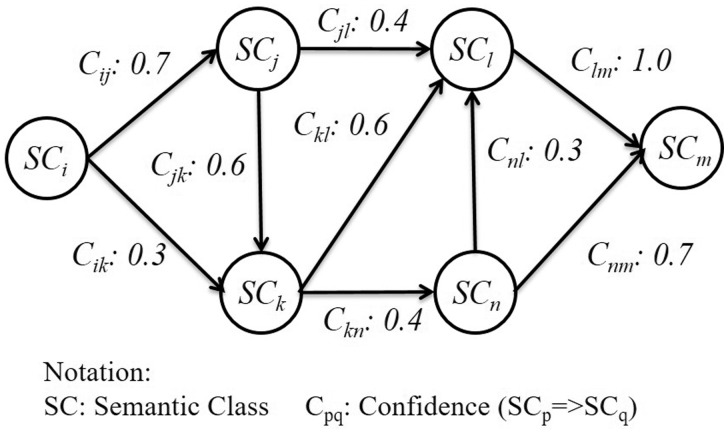
An interactive graph for pattern generation.

Figure 6 is an illustration of a semantic graph. In this graph, vertices (SC_*x*_) represent semantic classes; edges represent the co-occurrence of two classes, SC_*i*_ and SC_*j*_, where SC_*i*_ precedes SC_*j*_. The number on an edge denotes the confidence of two connecting vertices. After constructing all semantic graphs, we then generate interaction patterns by applying the random walk theory (27) in search of high frequency and representative classes for PPIs. Assuming we have a semantic graph *G* defined as *G *= (*V*, *E*) (|*V*|= *v*, |*E*|= *u*), a random walk process consists of a series of random selections on the graph. Every edge *E_nm_* has its own weight *M_nm_*, which denotes the probability of a semantic class SC_*n*_ followed by another class SC_*m*_. For each class, the sum of weights to all neighboring classes *N*(SC_*n*_) is defined as Equation (2), while the probability matrix of the graph is defined as Equation (3). A series of a random walk process now essentially becomes a Markov Chain. According to (28), the cover time of a random walk process on a normal graph is $\forall SCn,En\leq 4u^{2}$ with the selection of frequent SCs and their neighbors as the starting nodes of a random walk process. We conclude that with the use of random walk in finding frequent patterns on the interactive graph, we not only could capture combinations with low probability but also shorten the processing time.

(1) $$\begin{array}{l} \text{confidence}\left(\text{SC}i\Rightarrow \text{SC}j\right)=P\left(\text{SC}j|\text{SC}i\right)=\frac{\text{suppport}\left(\text{SC}i\cup \text{SC}j\right)}{\text{support}\left(\text{SC}i\right)}\text{, }\\ \text{where support}\text{min}=20,\text{ confidence}\text{min}=0.5 \end{array}$$

(2) ∀SCn∑m∈N(SCn)Mnm=1

(3) $$Pr=\left[Xt+1=\text{SC}m|\begin{array}{l} Xt=\text{SC}n\\ Xt-1=\text{SC}k\\ \;\ldots \\ X0=\text{SC}i \end{array}\right]=Pr\left[Xt+1=\text{SC}m|Xt=\text{SC}n\right]=M\text{nm}$$

Although the random walk process can help us capture representative interaction patterns in semantic graphs, it can also create some redundancy; a merging procedure is required to eliminate the redundant results by retaining patterns with long length and high coverage, and dispose of bigram patterns that could be covered by another pattern. For example, the pattern [*PROTEIN1*]- > [*Binding*] is completely covered by [*PROTEIN1*]- > [*Binding*]- > [*Regulation*]- > [*Transcription*]- > [*PROTEIN2*]; therefore we can incorporate the former with the latter. On the other hand, if a bigram pattern only partially matches another, the overlapping part is used as the pivot to concatenate the two patterns to form a longer pattern. For instance, [*Positive_regulation*]- > [*Regulation*] overlaps with [*Regulation*]- >  [*Gene_expression*]- > [*PROTEIN1*] on [*Regulation*], thus the two patterns are merged into a single pattern: [*Positive_regulation*]- > [*Regulation*]- >  [*Gene_ expression*]- > [*PROTEIN1*].

Reduction of SC labels through pattern selection is critical; it allows the successful execution of more sophisticated text classification algorithms, which leads to improved performance for PPI extraction. These algorithms cannot be executed on patterns before they are processed since redundant SC labels will result in excessively high execution time, making them impractical (26). To perform pattern selection, we use the log likelihood ratio (LLR) (26), an effective feature selection method to discriminate SCs for PPI instances. Given a training dataset comprised of positive instances, LLR employs Equation (4) to calculate the likelihood of the assumption that the occurrence of a semantic class *SC* in the expressions of PPI is not random,

(4) $$-2\log\left[\frac{p{\left(\text{SC}\right)}^{N(\text{SC}\wedge I)}{\left(1-p(\text{SC})\right)}^{N(I)-N(\text{SC}\wedge I)}p{\left(\text{SC}\right)}^{N(\text{SC}\wedge \neg I)}{\left(1-p(\text{SC})\right)}^{N(\neg I)-N(\text{SC}\wedge \neg I)}}{p{\left(\text{SC}|I\right)}^{N(\text{SC}\wedge I)}{\left(1-p(\text{SC}|I)\right)}^{N(I)-N(SC\wedge I)}p{\left(\text{SC}|\neg I\right)}^{N(SC\wedge \neg I)}{\left(1-p(\text{SC}|\neg I)\right)}^{N(\neg I)-N(\text{SC}\wedge \neg I)}}\right]$$

where *I* denotes the set of positive PPI sentences in the training dataset; *N*(*I*) and *N*(*¬I*) are the numbers of positive and negative PPI sentences, respectively; and *N*(*SC*∧*I*) is the number of positive PPI sentences containing the semantic class SC. The probabilities *p*(SC), *p*(SC*|I*), and *p*(SC*|¬I*) are estimated using maximum likelihood estimation. A SC with a large LLR value is thought to be closely associated with the interaction. Lastly, we rank the interaction patterns in the training dataset based on a summation of these semantic classes’ LLR values and retain the top 20 for representing PPIs. 

### IPT construction

**Figure 7.**
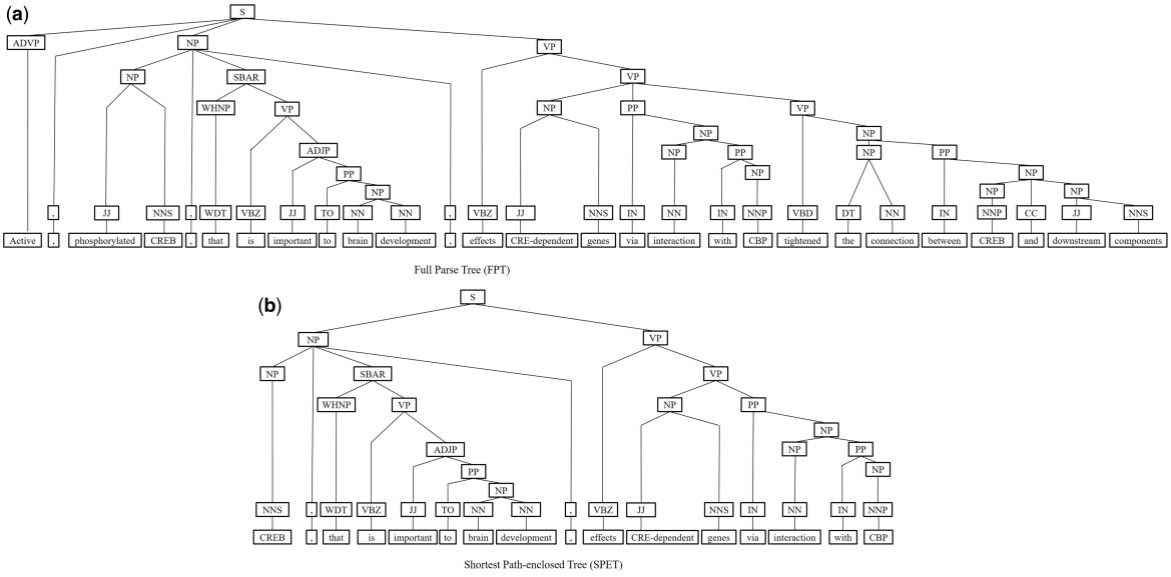
The FPT and SPET of the example sentence (a)FPT (b) SPET.

Next, we represent a candidate sentence by the proposed IPT structure, which is the SPET of a sentence enhanced by three operations: ‘branching’, ‘ornamenting’ and ‘pruning’. In reference (14), the authors show that SPET is effective in identifying the relation between two entities mentioned in a text sentence. Specifically, the SPET of a candidate sentence is the smallest sub-tree of the sentence’s syntactic parse tree that links target protein *p_i_* and *p_j_*. To show how we are improving SPET with IPT, we exemplify the operators by applying them to the sentence ‘Active, phosphorylated CREB, which is important to brain development, effects CRE-dependent genes via interaction with CBP which tightened the connection between CREB and downstream components.*’.* that expresses the interaction between ‘CREB’ and ‘CBP’. Figure 7a show the syntactic parse tree of the example sentence and the corresponding SPET is illustrated in Figure 7b. The three operations are described as follows.

**Figure 8.**
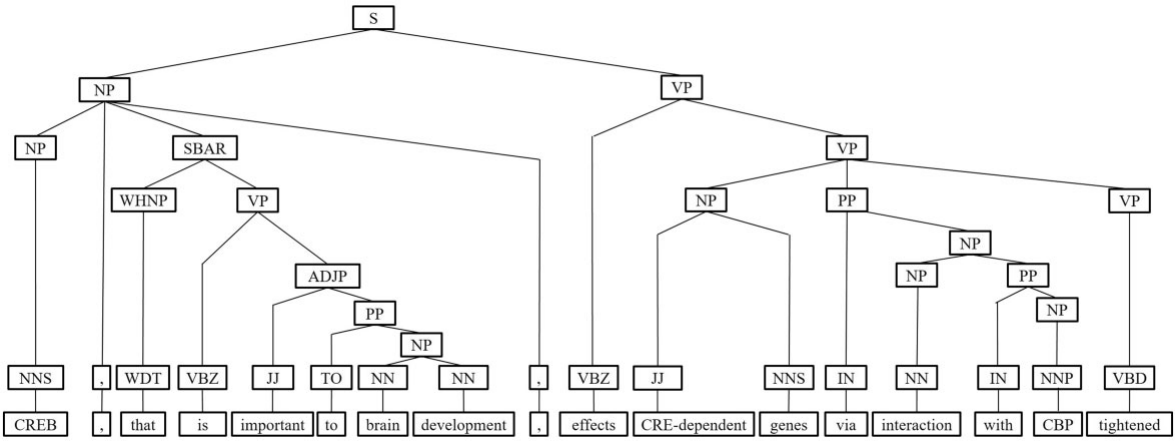
The branching operation of IPT of the example sentence.

*IPT branching.* Although Zhang *et al.* (29) demonstrated that the SPET is effective in identifying the relation between two entities mentioned in a textual sentence, the information in SPET is sometimes insufficient for detecting interaction between target proteins. For instance, in Figure 7a, the term ‘tightened’ and the corresponding syntactic constituent are critical for recognizing the interaction between CREB and CBP. However, they are excluded from the sentence’s SPET, as shown in Figure 7b. To include useful sentence context, the branching operator first examines the existence of verb behind the last target protein of the sentence. If a verb and the target protein form a verb phrase in the sentence’s syntactic parse tree, the verb is treated as a modifier of the target protein and is concatenated into the IPT. As shown in Figure 8, the branched IPT has included richer context information than its original.

*IPT pruning.* In later process we adopt SVM to classify interactive patterns. SVM is a vector space classification model that hypothesizes data of different classes form distinct contiguous regions in a high-dimensional vector space (25, 26); the hypothesis; however, is invalid if data representation is chosen improperly. We observed that IPTs would contain redundant elements that would influence the performance of interaction classification therefore we use the pruning operator to condense IPTs via the following procedures.

**Figure 9.**
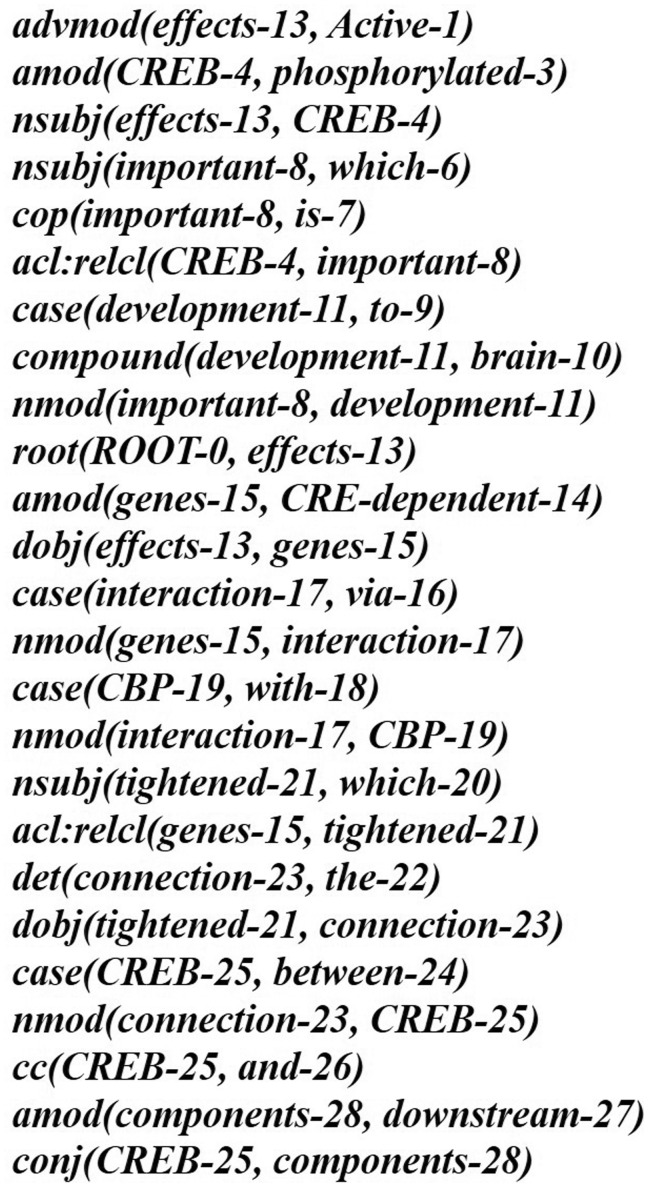
The typed dependencies representation of the example sentence.

**Figure 10.**
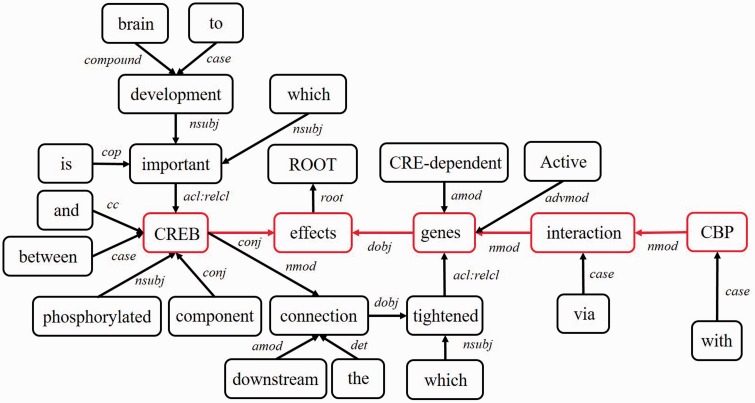
The dependency graph of the example sentence.

**Figure 11.**
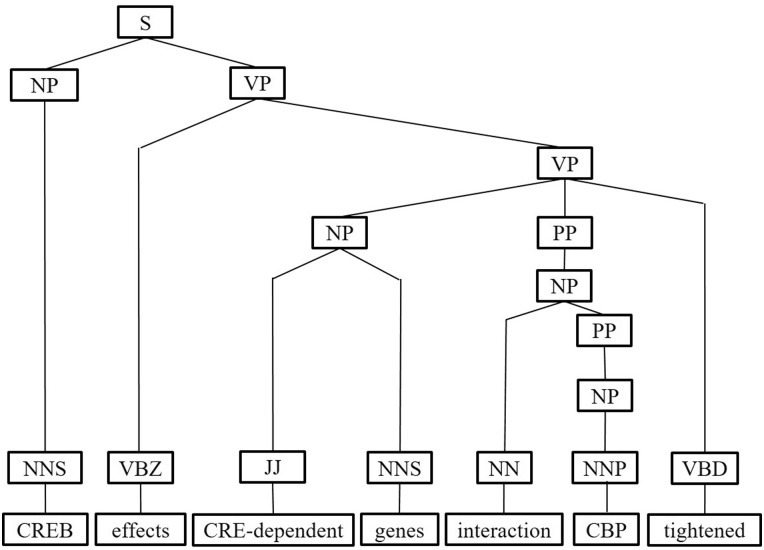
The pruning operation of IPT of the example sentence.
- Stop word removal: Frequent words are not useful for expressing interactions between proteins. For instance, the word ‘with’ in Figure 8 is a common preposition and cannot be utilized to discriminate interactive expressions. To remove stop words and the corresponding syntactic elements from the IPT, we sort words according to their frequency in the text corpus, and the most frequent words are used to compile a s*t*op w*o*rd list. More specifically, we selected the most frequent words whose accumulated frequencies reached 80% of the total word frequency count in the five corpora, since the rank-frequency distribution of words follows Zip’s law (26). Protein names and verbs are excluded from the list for refinement, since both are key constructs of protein-protein interactions.
- Duplicate element removal: Nodes in an IPT would be duplicated and therefore are redundant. A node is duplicated if it has a single child and its tag is also identical to that of its parent. For instance, the node VP in the last branch of Figure 8 is a duplicate node. Since the tree kernel we adopted to compute the similarity between text sentences is based on the percentage of overlap between IPTs, duplicate node*s* would degrade our system performance. To reduce their influen*c*e, the pruning operator deletes all duplicate nodes in an IPT. As shown in Figure 11, the pruned IPT is more concise and clearer than its original.

- Middle clause removal: Middle clauses of inter-clause candidate sentences may (or may not) be irrelevant to protein interactions. To discriminate middle clauses associated with the proteins, we adopted the Stanford parser (30) to label dependencies between text tokens (words). A labeled dependency is a triple of dependency name, governor token and dependent token. The labeled dependencies form a directed graph *G* = <*V*, *E*>, where each vertex in *V* is a token and the edges in *E* denote the set of dependencies. Figure 9 shows the dependencies extracted from the example sentence and the corresponding dependency graph is showed in Figure 10. Next, we search for the *protein dependency path* which we defined as the shortest connecting path of the target protein-pair in *G*. The example’s protein dependency path is highlighted in red in Figure 10 (CREB→conj effects ←dobj genes←nmod interaction ←nmod CBP). The pruning operator removes a middle clause and all its elements in IPT if the clause is not involved in the protein dependency path for the clause’s inability to make the target proteins associated. In Figure 11, the middle clause “that is important to brain development” is pruned because it is the complement of CREB, which is irrelevant to protein interactions.

**Figure 12.**
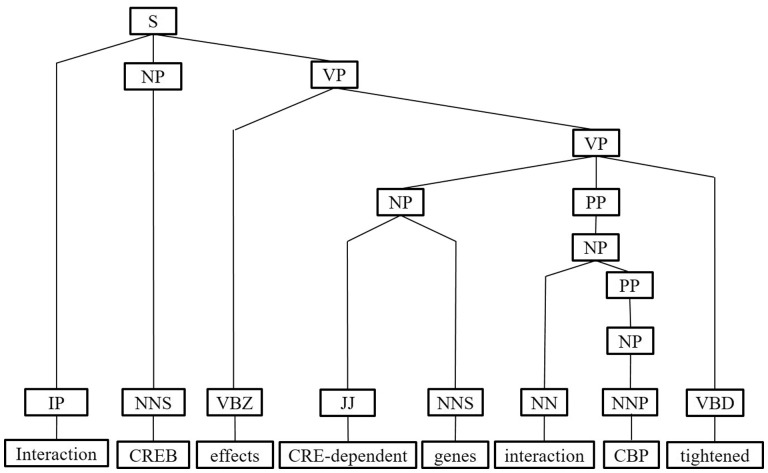
The ornamenting operation of IPT of the example sentence.

IPT ornamenting. Finally, the generated interaction patterns can help us capture the most prominent and representative patterns for expressing PPI. Highlighting interaction patterns closely associated with PPIs in an IPT would improve the interaction extraction performance. For each IPT that matched an interaction pattern, we add an IP tag as a child of the tree root to incorporate the interactive semantics into the IPT structure (as shown in Figure 12).

### Convolution tree kernel

Kernel approaches are frequently used in SVM to compute the dot product (i.e. similarity) between instances modeled in a complex feature space; here we employ the CTK (21) for measuring the similarity between sentences. A convolution kernel captures structured information in terms of substructures, hence we can represent a parse tree *T* by a vector of integer counts of each sub-tree type (regardless of its ancestors):

(5) ϕ(T)=(#subtree1(T),…,#subtreei(T),…,#subtreen(T)),

where *#*subtree_*i*_(*T)* is the occurrence number of the *i*th sub-tree type (subtree_*i*_) in *T*. Since the number of different sub-trees is exponential with the parse tree size, it is computationally infeasible to directly use the feature vector ϕ(T). To solve this computation issue, the CTK computes the syntactic similarity between the above high dimensional vectors implicitly as follows:

(6) $$\begin{array}{l} K\text{CTK}\left(T1|T2\right)=\langle \phi \left(T1\right)|\phi \left(T2\right)\rangle \\ \;=\sum i\#\text{subtree}i(T1)\cdot \text{\#subtree}i(T2)\\ \;=\sum i\left(\sum n1\in N1I1\text{subtree}i(n1)\right)\left(\sum n2\in N2I\text{subtree}i(n2)\right)\\ \;=\sum n1\in N1,n2\in N2\Delta \left(n1,n2\right) \end{array}$$

where *N_1_* and *N_2_* are the sets of nodes in trees *T_1_* and *T_2_*. Isubtreei(n) is a function whose value is 1 if there is a subtree_*i*_ rooted at node *n*, and zero otherwise. Specifically, the CTK *K_CTK_*considers the number of common sub-trees as the measurement of syntactic similarity between two interaction pattern trees *IPT_1_* and *IPT_2_* as follows:

(7) KCTK(IPT1, IPT2)=∑n1∈N1,n2∈N2Δ(n1,n2),

*N_1_* and *N_2_* are the sets of nodes in *IPT_1_* and *IPT_2_*, respectively. In addition Δ(*n*_1_, *n*_2_) evaluates the common sub-trees rooted at *n_1_* and *n_2_* and is computed recursively as follows:

- if the productions (i.e. the nodes with their direct children) at *n_1_* and *n_2_* are different, Δ(*n*_1_, *n*_2_) = 0;
- else if both *n*_1_ and *n*_2_ are pre-terminals (POS tags), Δ(*n*_1_, *n*_2_)=1×*λ;*
- else calculate Δ(n_1_, _n2_) recursively as:
  (8) Δ(n1,n2)=λ∏k=1#ch(n1)(1+Δ(ch(n1,k), ch(n2,k)))

wher*e #ch(n_1_)* is the number of children of node *n_1_*; *ch(n, k)* is the *k*th child of node *n*; and *λ*(0<*λ *< 1) is the decay factor used to make the kernel value less variable with respect to different sized sub-trees. The parse tree kernel counts the number of common sub-trees as the syntactic similarity measure between two PPI instances. The time complexity for computing this kernel isO(|N1|⋅|N2|) (21).

## Experiments

### Evaluation dataset

Due to the very recent completion of the BioCreative V BioC task, during edition of this article we have yet received the official annotation of the data used; therefore, we evaluated our method with five publicly available corpora that contain PPI annotations: LLL (31), IEPA (32), HPRD50 (33), AIMed (34) and BioInfer (35). AIMed, IEPA, HPRD50 and LLL were constructed specifically for PPI, while BioInfer is a more general-purpose corpus. All of them are commonly served as the standard corpora for training and testing PPI extraction programs. Specifically, AIMed contains 200 abstracts from PubMed that were identified as containing PPI by Database of Interacting Proteins [DIP (32)), from which the interactions between human genes and proteins in the abstracts were annotated manually. Additionally, certain abstracts that do not contain PPIs were added as negative examples. The current release of AIMed corpus is comprised of 225 of abstracts (10). BioInfer contains annotations for not only PPI but also other types of events. Pairs of interacting entities were extracted from DIP and used as query inputs to PubMed retrieval system, from which the returned abstracts were broken down into sentences; only sentences possessing more than one pair of interacting entities were kept. A random subset of the sentences was also annotated for entities of protein, gene and RNA relationships. After combining the above resultant sets into a PPI corpus, BioInfer consists of the maximum number of instances among the five corpora within 1100 sentences. In addition, IEPA was constructed of 486 sentences containing a specific pair of co-occurring chemicals from PubMed abstracts; the interactions between pairs of entities were annotated while the majority of the entities were proteins. Unlike the above corpora, HPRD50 was constructed by taking 50 random abstracts referenced by the Human Protein Reference Database [HPRD (33)]. Human proteins and genes were identified by ProMiner (36) software, while direct physical interactions, regulatory relations as well as modifications were annotated by experts. The corpus was developed as a test set for the RelEx (33) system, containing 145 sentences with annotations and lists of positive/negative PPI. LLL corpus was originally created for the Learning Language in Logic 2005 (LLL05) challenge, a task to learn rules to extract protein/gene interactions from biology abstracts from the Medline bibliography database. It contains three types of gene interaction of Bacillus subtilis: explicit action, binding of a protein on the promoter of the target gene, and membership in a regulon family (10). The corpus, albeit serving as an independent test set, contains only 77 sentences, making it the smallest dataset among the five corpora.

### Experimental setting and evaluation methods

The description of the corpora is shown in Table 1; both the size and the distribution of positive/negative elements are shown. All corpora are parsed using the Stanford parser (http://nlp.stanford.edu/software/lex-parser.shtml) to generate the output of parse tree and POS tagging. For our implementation, we use the Moschitti’s tree kernel toolkit (22) to develop the convolution kernel of an IPT. Following conventions, we set the parameters C for SVM to the ratio of negative instance to positive ones in respective corpora, and *λ* for the CTK to default 0.4 (18, 19, 29). To derive credible evaluation results, we utilize various performance measures on all of the corpora including cross-validation (CV) (26), cross-learning (CL) (10) and cross-corpus (CC) (37). This guarantees the maximal use of the available data and allows comparison to the previous relevant research results. In the setting of CV, we trained and tested each method on the same corpus using 10-fold CV. Although the 10-fold CV has become the de facto standard of PPI extraction evaluation, it is also somewhat biased due to the fact that training and the test data sets are similar in nature. Since the ultimate goal of PPI extraction is the identification of PPIs in biomedical texts with unknown characteristics, we performed experiments with learning across corpora, where the training and test data sets are drawn from different distributions. For the CL experiments, each corpus was selected as the testing set once, where ensemble of four remaining corpora was used for training. Finally, for the CC experiments, we trained the model on one corpus and tested it on each of the other four corpora separately. The evaluation metrics are precision, recall and F_1_-measure (26), as well as the micro-average used for comparing the average performance. These measures are defined based on a contingency table of predictions for a target corpus *C_k_*. The precision *P*(*C_k_*), recall *R*(*C_k_*), F_1_-measure *F_1_*(*C_k_*) and micro-average *F^μ^* are defined as follows:

(9) $$P(Ck)=\frac{\text{TP}(Ck)}{\text{TP}(Ck)+\text{FP}(Ck)}$$

(10) $$R(Ck)=\frac{\text{TP}(Ck)}{\text{TP}(Ck)+\text{FN}(Ck)}$$

(11) $$F1(Ck)=\frac{2\times P(Ck)\times R(Ck)}{P(Ck)+R(Ck)}$$

(12) $$F^{\mu }=\frac{\sum_{k=1}^{n}2\times P(Ck)\times R(Ck)}{\sum_{k=1}^{n}P(Ck)+R(Ck)}$$

**Table 1.** Distribution of the five corpora used for performance evaluation of PPI extraction.

Statistics	Corpora				
	LLL	IEPA	HPRD50	AIMed	BioInfer
no. POS.	164	335	163	1000	2534
no. NEG.	166	482	270	4834	7132
no. sentence	77	486	145	1955	1100

*TP*(*C_k_*) denotes the number of true positives, i.e. the number of positive instances that are correctly classified. The *FP*(*C_k_*) denotes the number of false positives, which are negative instances that are erroneously classified as positives. Analogously, *TN*(*C_k_*) and *FN*(*C_k_*) stand for the number of true negatives and false negatives, respectively. The F_1_ value is used to determine relative effectiveness of the compared methods.

## Results and Discussion

For our CV experiment, the proposed IPT structure uses three operators, ‘branching, pruning’ and ‘ornamenting’, to enhance SPET. In the following, we evaluate the performance of these operators to demonstrate the effectiveness of IPT. Table 2 shows the marginal performances in 10-fold cross-validation of applying IPT branching, pruning and ornamenting, denoted as +IPT_branching_, + IPT_pruning_ and +IPT_ornamenting_ respectively. As shown in the table, IPT branching (i.e. +IPT_branching_) outperforms SPET because branching operator correctly incorporated extra context information to remedy the context-limited problem of the SPET (see Section ‘IPT construction’). The pruning operator further improves the system performance for successfully eliminating indiscriminative and redundant IPT elements and thereby helps SVM learn representative syntactic structures of PPIs. Notably, the IPT ornamenting operator improves the F_1_ performance significantly, because generated interaction patterns are highly correlated with PPIs. Thus, tagging them in the IPT structure helps our method discriminate PPI passages. As the operators polish the SPET from different perspectives without having conflicts with one another. Consequently, applying the operators altogether achieves the best performance.

**Table 2.** Incremental contribution of the IPT branching, pruning and ornamenting operators

**IPT** **structure**	LLL	IEPA	HPRD50	AIMed	BioInfer	*F^μ^*
	
**Precision, recall, F_1_-measure (%)**						
SPET	56.4/96.1/69.6	55.5/28.8/37.1	46.2/13.4/20.8	29.2/39.9/32.4	26.7/**89.4**/41.0	30.0/67.8/41.6
+IPT_branching_	58.3/**96.3**/72.6	58.3/42.6/49.2	56.3/38.9/46.0	38.3/46.4/42.0	42.3/81.8/55.8	42.5/68.1/52.3
+IPT_pruning_	62.3/93.5/74.8	58.9/60.5/59.7	59.1/63.9/61.4	49.6/55.8/52.5	55.6/73.8/63.4	54.1/67.7/60.2
+IPT_ornamenting_	**73.2**/89.6/**80.6**	**62.5**/**83.3**/**71.4**	**63.8**/**81.2**/**71.5**	**57.2**/**64.5**/60.6	**68.6**/70.3/**69.4**	**64.4/69.6/66.9**

The proposed IPT kernel uses the PPI patterns to enhance the SPET, and it is compared with several feature-based, kernel-based and multiple kernel PPI extraction methods mentioned in related work to demonstrate its effectiveness. As shown in Table 3, the proposed method significantly outperforms AkanePPI. Furthermore, the syntax tree-based kernel methods (i.e. ST, SST, PT and SpT) only examine the syntactic structures within texts but cannot sense the semantics of protein interactions. In contrast, our method analyzes the semantics and contents (i.e. PPI patterns) within the text to identify PPIs, making its performance superior to those of the syntax tree-based kernel methods. It is noteworthy that the syntax tree-based kernel methods are at times only on par with the co-occurrence approach in terms of F_1_-measure. This can be observed on the relatively small corpus LLL, in which their results practically coincide with the co-occurrence method. On the other hand, PIPE delivers good result on both precision and F_1_-measure in a broader corpus such as BioInfer. The RFB and Cosine method also outperform SPET, AkanePPI and syntax tree-based kernel methods as they incorporate dependency features to distinguish PPIs. Nevertheless, although the Cosine method can accomplish higher performance by further considering term weighting, it is difficult to demonstrate word relations through symbolic representations in this approach. On the contrary, our method can extract word semantics and generate PPI patterns that can capture relations between distant-located mentions in the text; consequently we can achieve comparable outcome. SL, GK and CK approaches outperform our method because their hybrid kernels can adequately encapsulate information required for relation prediction based on sentential structures involved in two entities; nonetheless, our method is able to capture more PPI instances through the acquired PPI patterns. Thus, we can achieve higher recall than both CK-based approaches on all five corpora, which leads to a comparable overall performance.

**Table 3.** CV results of the compared methods

System	LLL	IEPA	HPRD50	AIMed	BioInfer	*F^μ^*
	
**Precision,** **recall, F_1_-measure (%)**						
SPET	56.4/96.1/69.6	55.5/28.8/37.1	46.2/13.4/20.8	29.2/39.9/32.4	26.7/89.4/41.0	30.0/67.8/41.6
AkanePPI	76.7/40.2/52.8	66.2/51.3/57.8	52.0/55.8/53.8	29.1/52.9/37.5	56.8/85.4/68.2	48.1/71.0/57.3
Co-occurrence	55.9/**100.**/70.3	40.8/**100.**/57.6	38.9/**100.**/55.4	17.8/**100.**/30.1	26.6/**100.**/41.7	25.2/**100.**/40.2
PT	56.2/97.3/69.3	63.1/66.3/63.8	54.9/56.7/52.4	39.2/31.9/34.6	45.3/58.1/50.5	44.5/50.3/47.2
SST	55.9/**100.**/70.3	54.8/76.9/63.4	48.1/63.8/52.2	42.6/19.4/26.2	47.0/54.3/50.1	46.1/44.6/45.3
ST	55.9/**100.**/70.3	59.4/75.6/65.9	49.7/67.8/54.5	40.3/25.5/30.9	46.8/60.0/52.2	45.4/49.9/47.6
SpT	55.9/**100.**/70.3	54.5/81.8/64.7	49.3/71.7/56.4	33.0/25.5/27.3	44.0/68.2/53.4	41.1/54.9/47.0
RFB^a^	72.0/73.0/73.0	64.0/70.0/67.0	60.0/51.0/55.0	49.0/44.0/46.0	−/-/-	
edit	68.0/98.0/78.4	**77.2**/60.2/67.1	71.3/45.2/53.3	**68.8**/27.7/39.0	50.4/39.2/43.8	58.8/37.6/45.9
Cosine	70.2/81.7/73.8	61.3/68.4/64.1	59.0/67.2/61.2	43.6/39.4/40.9	44.8/44.0/44.1	46.0/44.8/45.4
SL	69.0/85.3/74.5	69.5/71.2/69.3	64.4/67.0/64.2	47.5/65.5/54.5	55.1/66.5/60.0	53.7/66.8/59.5
GK	72.5/87.2/76.8	69.6/82.7/**75.1**	64.3/65.8/63.4	52.9/61.8/56.4	56.7/67.2/61.3	56.5/66.4/61.1
CK	**77.6**/86.0/80.1	67.5/78.6/71.7	**68.5**/76.1/70.9	55.0/68.8/**60.8**	65.7/71.1/68.1	62.4/71.1/66.5
PIPE	73.2/89.6/**80.6**	62.5/83.3/71.4	63.8/81.2/**71.5**	57.2/64.5/60.6	**68.6**/70.3/**69.4**	**64.4/**69.6**/66.9**

a Since the original work did not include experiment on BioInfer, we therefore leave out *F^μ^* in the table.

Table 4 lists our results regarding the CL performances. Five additional methods were used in comparison with our proposed method. First, it is interesting to note that while the SPET had a F_1_-measure of 41.6% in the CV setting, it showed a decrease by 12% in the CL setting due to the lower performance in AIMed and BioInfer; SpT, Cosine and edit methods too suffered a significant drop in their performance. SpT achieved rather poor performance in this scenario, especially on the IEPA corpus. It obtained a very low score due to the extremely low recall. The Cosine and edit method were on par with SpT, each of which surpassed the other two in certain corpora. The SL kernel showed a modest drop on the average F_1_-measure by about 6%, and demonstrated a relatively consistent performance across all five corpora in terms of the major evaluation measures. Finally, our method exhibited the highest stability, with each and every case under the CL setting outperforming those of the CV results. The overall performance of our IPT kernel is improved with the CL setting, and also outperformed all other methods on the five corpora.

**Table 4.** CL results of the compared methods

System	LLL	IEPA	HPRD50	AIMed	BioInfer	*F^μ^*
	
Precision, recall, F_1_-measure (%)						
SPET	74.5/48.2/58.5	**58.8**/31.9/41.4	58.2/39.3/46.9	28.9/27.8/28.3	33.9/21.0/26.0	34.8/24.8/29.0
SpT	48.2/83.5/61.2	41.6/19.6/15.5	44.7/77.3/56.6	20.3/48.4/28.6	38.9/48.0/43.0	33.0/48.2/39.2
edit	68.1/48.2/56.4	58.1/45.1/50.8	58.1/55.2/56.6	26.8/59.7/37.0	53.0/22.7/31.7	44.7/37.7/40.9
Cosine	**80.3**/37.2/50.8	46.3/31.6/37.6	**63.0**/56.4/59.6	27.5/59.1/37.6	42.1/32.2/36.5	38.6/42.1/42.3
SL	79.0/57.3/66.4	71.0/52.5/60.4	56.9/68.7/62.2	28.3/**86.6**/42.6	62.8/36.5/46.2	51.6/55.6/53.5
PIPE	68.6/**90.7**/**78.1**	55.6/**87.2**/**67.9**	62.7/**80.1**/**70.3**	**52.9**/61.2/**56.7**	**63.3**/**64.6**/**63.9**	**59.5/65.4/62.3**

Due to the existing variety of the nature of the five corpora, such as the types of named entities annotated, the definition of what exactly constitutes an interaction, and the relative positive/negative distributions of relation pairs, we conducted a CC evaluation to shed light on whether the learned models can be generalized beyond the specific characteristics of the training data. Table 5 shows the CC results, in which different methods were trained on one corpus, and subsequently tested on the four remaining corpora. The rows and columns correspond to the training and test corpora, respectively. Cross-validated results were enclosed in parentheses for reference in the table, and bold-texted results indicate the best overall result for a particular corpus. We speculated that the average CC performances would be worse than the CL performances due to the smaller size of the training dataset; however, the CC results did not completely substantiate our hypothesis. For instance, PIPE achieves 71.3% F_1_-measures on HPRD50 trained on the BioInfer, whereas it achieves only 70.3% when trained using the combination of other four corpora. In general, the overall performance of our method is superior to those of other kernel-based methods. Specifically, the model tested on the HPRD50 corpus achieved F_1_-measures of 69.3, 67.8, 69.4 and 71.3% when trained on the LLL, IEPA, AIMed and BioInfer, respectively. It outperformed all other approaches using the same training dataset. This is because the PPI patterns generated successfully captured the accurate PPIs. For instance, the interaction pattern ‘[PROTEIN1]- > [Positive_regulation]- > [PROTEIN2]- > [Negative_regulation]’ generated from the HPRD50 corpus is capable of matching the positive instance ‘Amyloid beta protein stimulation of phospholipase C was absent from LA-N-2 cells previously treated with norepinephrine, trans-1-amino-1,3-cyclopentanedicarboxylic acid, bombesin, or amyloid beta peptide’ in the IEPA corpus, which describes the interaction between the protein ‘Amyloid beta protein’ and ‘phospholipase C’. In addition, our method trained on the IEPA corpus achieved comparable performances to that of the CK when tested on LLL and HPRD50. This also demonstrates that the generated PPI patterns from our method of IEPA are effective in matching positive instances of the tested corpora. For instance, the generated interaction pattern ‘[*PROTEIN1*]- > [*Negative_regulation*]- > [*PROTEIN2*]- > [*Localization*]’ from the IEPA corpus is able to capture texts such as ‘*Both leptin and insulin can reduce hypothalamic NPY production and secretion*’, in which ‘leptin’ and ‘NPY’ represent PROTEIN1 and PROTEIN2, respectively. On the other hand, the performance of our method is slightly inferior to both multi-kernel-based approaches when trained on the smallest LLL corpus. More specifically, when trained on larger corpora (IEPA and HPRD50), our method can generate more extensive PPI patterns, leading to a broader coverage and hence a higher recall. As a result, our method is more effective than the others, since the generated PPI patterns can retrieve more information within PPIs.

**Table 5.** CC results of the compared methods with F_1_-measure

System	Training corpus	LLL	IEPA	HPRD50	AIMed	BioInfer	*F^μ^*
		
		F_1_-measure (%)					
SPET	LLL	(69.6)	63.0	57.0	23.3	39.9	36.3
	IEPA	55.9	(37.1)	47.8	25.3	37.2	33.8
	HPRD50	48.8	38.6	(20.8)	26.2	35.1	32.1
	AIMed	28.3	26.2	18.6	(32.4)	32.0	31.4
	BioInfer	30.5	27.7	17.5	26.9	(41.0)	34.7
PT	LLL	(69.3)	55.2	55.5	30.0	43.3	40.1
	IEPA	43.5	(63.8)	43.0	28.4	19.8	25.9
	HPRD50	40.7	57.1	(52.4)	35.8	29.7	33.9
	AIMed	13.6	25.2	47.9	(34.6)	17.8	24.6
	BioInfer	60.2	25.6	38.6	34.6	(50.5)	43.8
SST	LLL	(70.3)	58.2	54.7	29.3	41.5	39.0
	IEPA	56.6	(63.4)	53.1	32.7	29.9	33.6
	HPRD50	49.3	57.2	(52.2)	31.6	38.0	37.3
	AIMed	11.4	01.2	24.2	(26.2)	13.3	17.4
	BioInfer	65.0	36.8	37.2	34.1	(50.1)	44.0
ST	LLL	(70.3)	61.7	53.8	32.4	47.9	43.8
	IEPA	43.1	(65.9)	41.4	33.1	44.2	41.4
	HPRD50	44.7	52.8	(54.5)	38.2	39.9	40.4
	AIMed	12.4	33.9	45.8	(30.9)	21.4	25.7
	BioInfer	58.1	23.5	30.5	33.5	(52.2)	44.0
SpT	LLL	(70.3)	59.0	54.7	31.7	50.3	44.9
	IEPA	54.4	(64.7)	49.0	32.7	31.1	34.2
	HPRD50	49.3	55.4	(56.4)	32.5	40.0	38.8
	AIMed	17.2	18.6	43.2	(27.3)	24.3	25.4
	BioInfer	58.2	56.4	51.3	34.7	(53.4)	47.2
Cosine	LLL	(73.8)	63.8	56.0	30.2	42.6	40.3
	IEPA	66.5	(64.1)	53.5	30.1	41.4	39.4
	HPRD50	53.0	45.5	(61.2)	32.7	37.7	37.3
	AIMed	32.7	23.0	51.2	(40.9)	30.3	34.1
	BioInfer	64.7	43.6	63.0	36.5	(44.1)	42.4
edit	LLL	(78.4)	63.1	61.5	32.0	44.7	42.3
	IEPA	64.3	(67.1)	52.4	34.0	38.5	39.2
	HPRD50	47.5	41.9	(53.3)	39.4	31.7	35.7
	AIMed	03.6	07.5	38.3	(39.0)	15.9	23.7
	BioInfer	60.8	58.4	62.4	39.6	(43.8)	43.9
SL	LLL	(74.5)	62.6	59.0	32.4	47.9	44.1
	IEPA	66.4	(69.3)	58.2	33.1	44.2	42.4
	HPRD50	61.4	53.3	(64.2)	38.2	39.9	41.0
	AIMed	36.5	25.5	59.0	(54.5)	29.2	38.6
	BioInfer	74.6	64.0	61.8	41.5	(66.5)	57.9
GK	LLL	(76.8)	64.9	59.8	33.3	42.5	41.5
	IEPA	77.6	(75.1)	67.5	39.1	51.7	49.4
	HPRD50	77.6	65.1	(63.4)	42.2	42.5	44.7
	AIMed	74.5	67.4	69.0	(56.4)	47.1	52.3
	BioInfer	78.0	68.0	63.9	47.2	(61.3)	57.2
CK	LLL	(80.1)	65.6	64.0	38.6	48.9	47.2
	IEPA	83.2	(71.7)	66.5	40.4	55.8	52.1
	HPRD50	72.2	67.8	(70.9)	43.9	48.6	48.9
	AIMed	73.5	68.1	68.3	(60.8)	53.1	57.2
	BioInfer	76.9	71.4	68.3	49.6	(68.1)	62.1
PIPE	LLL	(80.6)	65.1	69.3	42.3	51.9	**50.2**
	IEPA	84.5	(71.4)	67.8	44.5	62.1	**57.1**
	HPRD50	72.1	68.2	(71.5)	46.8	53.3	**52.6**
	AIMed	75.2	69.0	69.4	(60.6)	58.2	**60.1**
	BioInfer	78.5	72.3	71.3	52.1	(69.4)	**63.9**

Bold typeface indicates our best overall result for a corpus (differences under 1 base point are ignored).

Note that the evaluation results using other corpora are no better than those from internal 10-fold CV. This is because the annotation policies are different, and the classifiers cannot predict these differences. The model based on an original corpus performs better than the models based on other corpora in other cases, but the results are up to 7.3% better F1-score than for the best performing model based on other corpora. However, the results on the LLL corpus using classifiers trained on IEPA are better than the 10-fold CV result using LLL corpus itself for training. Based on our further analysis, we conclude that IEPA and LLL are very similar regarding PPI. Thus, learning with IEPA is more robust than 10-fold CV within LLL. It is interesting to note that PIPE is able to perform well when trained on IEPA, which is much smaller than AIMed and BioInfer. In general, learning with larger corpora produces better performance. Nevertheless, the better annotation quality of IEPA enables PIPE to learn discriminative interaction patterns.

Based on our preliminary observations, PIPE is able to achieve comparable performance on BioC corpus, which contains mostly full-text articles. This is because a relatively high proportion of PPI passages are short, and PIPE can thus capture interaction expressions. For instance, the candidate segment ‘We have identified a third Sec24p family member also known as Iss1p, as a protein that binds to Sec16p.’ is correctly recognized as PPI passage due to successful match of generated interaction pattern ‘[PROTEIN1]- > [Binding]- > [PROTEIN2]- > [Negative_ regulation]’. However, based on our further analysis of the detection performance, our approach cannot effectively deal with longer candidate segments. For one, PIPE incorrectly classifies ‘Chromosomal deletion of LST1 is not lethal, but inhibits transport of the plasma membrane proton-ATPase (Pma1p) to the cell surface, causing poor growth on media of low pH’ as a PPI passage. This is because it is possible that the long text segments in the syntactic structures were so complex that they confused the dependency parsing process. As a result, the generated protein dependency paths were prone to errors that affected the accuracy of the removed middle clause and the corresponding extraction performance. In addition, we paired proteins in order to enumerate text segments that may convey PPIs; nevertheless, the issue of coreference resolution is not considered in this article as related studies are still in progress (38, 39). Therefore, a relatively low proportion of PPI passages cannot be captured by the candidate segment generation algorithm if the target protein name is referred to by a pronoun. We acknowledge this as an important issue for future research.

In summary, the proposed IPT kernel approach is able to generated discriminative interaction patterns that can describe the syntactic and semantic relations within a PPI expression and assist in detecting the interactions. We consider it as the foundation for a more profound understanding of the PPI structures to enhance the SPET. This method not only outperforms feature-based and kernel-based approaches, but also achieves comparable performances to those of multi-kernel-based methods. In addition, the patterns are easily interpretable by humans, and can be considered as the fundamental knowledge in understanding PPI expressions.

## Concluding remarks

Automated extraction of PPIs is an important and widely studied task in biomedical text mining. To this end, we proposed an interaction pattern generation approach for acquiring PPI patterns, which was utilized in the Collaborative Biocurator Assistant Task at BioCreative V. We also developed a method that combines the SPET structure with the generated PPI patterns to analyse the syntactic, semantic and context information in text. It then exploits the derived information to identify PPIs in biomedical literatures. Our experiment results demonstrate that the proposed method is effective and also outperforms well-known PPI extraction methods.

In the future, we will investigate other aspects, such as the dependency construction in texts, to incorporate even deeper semantic information into the IPT structures. We will also utilize information extraction algorithms to extract interaction tuples from positive instances and construct an interaction network of proteins.

## References

[1] Tikk D. Thomas P. Palaga P. (2010) A comprehensive benchmark of Kernel methods to extract protein–protein interactions from literature. PLoS Comput. Biol., 6, 1–19. 10.1371/journal.pcbi.1000837 PMC2895635 20617200

[2] Li L. Guo R. Jiang Z. Huang D. (2015) An approach to improve kernel-based protein protein interaction extraction by learning from large-scale network data. Methods, 83, 44–50. 25864936 10.1016/j.ymeth.2015.03.026

[3] López Y. Nakai K. Patil A. (2015) HitPredict version 4: comprehensive reliability scoring of physical protein-protein interactions from more than 100 species. Database, 2015, 10.1093/database/bav117 PMC4691340 26708988

[4] Phizicky E.M. Fields S. (1995) Protein-protein interactions: methods for detection and analysis. Microbiol. Rev., 59, 94–123. 7708014 10.1128/mr.59.1.94-123.1995 PMC239356

[5] Kim S. Islamaj Dogan R. Chatr-Aryamontri A. (2016) BioCreative V BioC Track Overview: Collaborative Biocurator Assistant Task for BioGRID. Database, 10.1093/database/baw121 PMC5009341 27589962

[6] Comeau D.C. Islamaj Dogan R. Ciccarese P. (2013) BioC: a minimalist approach to interoperability for biomedical text processing. Database, Vol. 2013. bat064. 10.1093/database/bat064 PMC3889917 24048470

[7] Airola A. Pyysalo S. Björne J. (2008) All-paths graph kernel for protein-protein interaction extraction with evaluation of cross-corpus learning. BMC Bioinformatics, 9, S2. 19025688 10.1186/1471-2105-9-S11-S2 PMC2586751

[8] Landeghem S.V. Saeys Y. Baets B. Peer Y.V. (2008) Extracting protein-protein interactions from text using rich feature vectors and feature selection. In: *Proceedings of 3rd International Symposium on Semantic Mining in Biomedicine*, pp. 77–84.

[9] Satre R. Sagae K. Tsujii J. (2007) Syntactic features for protein-protein interaction extraction. In: *Proceedings of the 2nd International Symposium on Languages in Biology and Medicine*, pp. 6.1–6.14.

[10] Pyysalo S. Airola A. Heimonen J. (2008) Comparative Analysis of Five Protein-protein Interaction Corpora. BMC Bioinformatics, 9, S6. 18426551 10.1186/1471-2105-9-S3-S6 PMC2349296

[11] Moschitti A. (2006) Efficient convolution kernels for dependency and constituent syntactic trees. In: *Proceedings of the 17th European Conference on Machine Learning*, pp. 318–329.

[12] Vishwanathan S.V.N. Smola A.J. (2002) Fast kernels for string and tree matching. In: *Proceedings of Neural Information Processing Systems*, pp. 569–576.

[13] Kuboyama T. Hirata K. Kashima H. (2007) A spectrum tree kernel. Inform. Media Technol., 2, 292–299.

[14] Zhang M. Zhou G.D. Aw A.T. (2008) Exploring syntactic structured features over parse trees for relation extraction using kernel methods. Inform. Process. Manage., 44, 687–701.

[15] Giuliano C. Lavelli A. Romano L. (2006) Exploiting shallow linguistic information for relation extraction from biomedical literature. In: *Proceedings of the 11th Conference of the European Chapter of the Association for Computational Linguistics* pp. 401–408.

[16] Miwaa M. Sætre R. Miyao Y. Tsujii J. (2009) Protein–protein interaction extraction by leveraging multiple kernels and parsers. Int. J. Med. Inform., 78, 39–46. 19501018 10.1016/j.ijmedinf.2009.04.010

[17] Cristianini N. Taylor J.S. (2000) An Introduction to Support Vector Machines and Other kernel-Based Learning Methods. Cambridge University Press, New York, USA.

[18] Qian L. Zhou G. (2012) Tree kernel-based protein–protein interaction extraction from biomedical literature. J. Biomed. Inform., 45, 535–543. 22388011 10.1016/j.jbi.2012.02.004

[19] Yang Z. Tang N. Zhang X. (2011) Multiple kernel learning in protein-protein interaction extraction from biomedical literature. Artif. Intell. Med., 51, 163–173. 21208788 10.1016/j.artmed.2010.12.002

[20] Erkan G. Özgür A. Radev D.R. (2007) Semi-supervised classification for extracting protein interaction sentences using dependency parsing. In: *Proceedings of the 2007 Joint Conference on Empirical Methods in Natural Language Processing and Computational Natural Language Learning*, pp. 228–237.

[21] Collins M. Duffy N. (2001) Convolution kernels for natural language. In: *Proceedings of Annual Conference on Neural Information Processing Systems*, pp. 625–632.

[22] Moschitti A. (2004) A study on convolution kernels for shallow semantic parsing. In: *Proceedings of the 42nd Annual Meeting of the Association for Computational Linguistics*, pp. 21–26.

[23] Porter M.F. (1997) An algorithm for suffix stripping In: J Karen Sparck W Peter (ed). Readings in Information Retrieval. Morgan Kaufmann, San Francisco.

[24] Kim J.D. Ohta T. Pyysalo S. (2009) Overview of BioNLP'09 shared task on event extraction. In: *Proceeding of the Workshop on Current Trends in Biomedical Natural Language Processing: Shared Task*, pp. 1–9.

[25] Han J. Kamber M. Pei J. (2011) Data Mining: Concepts and Techniques, 3rd edn Morgan Kaufmann, Waltham, Massachusetts.

[26] Manning C.D Schütze H. (1999) Foundations of Statistical Natural Language Processing, 1st edn MIT Press, Cambridge, Massachusetts.

[27] Lovász L. (1993) Random Walks on Graphs: A Survey. Janos Bolyai Mathematical Society, Budapest, pp. 1–46.

[28] Cooper C. Frieze A.M. (2005) The cover time of random regular graphs. SIAM J. Discr. Math., 18, 728–740.

[29] Zhang M. Zhang J. Su J. Zhou G.D. (2006) A composite kernel to extract relations between entities with both flat and structured features. In: *Proceedings of the 21st International Conference on Computational Linguistics and the 44th Annual Meeting of the Association for Computational Linguistics*, pp. 825–832.

[30] Marneffe M. MacCartney B. Manning C.D. 2006 Generating typed dependency parses from phrase structure parses. In: *LREC 2006*.

[31] Nedellec C. (2005) Learning language in logic-genic interaction extraction challenge. In: *Proceedings of the Learning Language in Logic 2005 Workshop at the International Conference on Machine Learning*, pp. 97–99.

[32] Xenarios I. Fernandez E. Salwinski L. (2001) DIP: The database of interacting proteins: 2001 update. Nucleic Acids Res., 29, 239–241. 11125102 10.1093/nar/29.1.239 PMC29798

[33] Fundel K. Küffner R. Zimmer R. (2007) RelEx - relation extraction using dependency parse trees. Bioinformatics, 23, 365–371. 17142812 10.1093/bioinformatics/btl616

[34] Bunescu R.C. Ge R. Kate R.J. (2005) Comparative experiments on learning information extractors for proteins and their interactions. Artif. Intell.Med., 33, 39–55. 10.1016/j.artmed.2004.07.016 15811782

[35] Pyysalo S. Ginter F. Heimonen J. (2007) A corpus for information extraction in the biomedical domain. BMC Bioinformatics, 8, 50–74. 17291334 10.1186/1471-2105-8-50 PMC1808065

[36] Hanisch D. Fundel K. Mevissen H.T. (2005) Prominer: rule-based protein and gene entity recognition. BMC Bioinformatics, 6, S14. 15960826 10.1186/1471-2105-6-S1-S14 PMC1869006

[37] Kabiljo R. Clegg A. Shepherd A. (2009) A realistic assessment of methods for extracting gene/protein interactions from free text. BMC Bioinformatics, 10, 233–245. 19635172 10.1186/1471-2105-10-233 PMC2723093

[38] Kamune K.P. Avinash A. (2015) Hybrid approach to pronominal anaphora resolution in English newspaper text. Int. J. Intell. Syst. Appl., 7, 56.

[39] Jonnalagadda S.R. Li D. Sohn S. (2012) Coreference analysis in clinical notes: a multi-pass sieve with alternate anaphora resolution modules. J. Am. Med. Inform. Assoc., 19, 867–874. 22707745 10.1136/amiajnl-2011-000766 PMC3422831

